# Supplementary material for: Picogram-Level Nanoplastic Analysis with Nanoelectromechanical System Fourier Transform Infrared Spectroscopy: NEMS-FTIR
Source: ACS Nano. 2026 Apr 1;20(14):11193–208. doi: 10.1021/acsnano.5c22099 (PMC13085850; doi:10.1021/acsnano.5c22099)
Supplement: Supplementary file 1 [file nn5c22099_si_001.pdf]

Supporting Information

Picogram-Level Nanoplastic Analysis with Nanoelectromechanical System Fourier Transform Infrared Spectroscopy: NEMS-FTIR

Jelena Timarac-Popović,[*,†,‡] Johannes Hiesberger,[‡] Eldira Šesto,[†] Niklas Luhmann,[‡] Ariane Giesriegl,[†] Hajrudin Bešić,[†,‡] Josiane P. Lafleur,[‡] and Silvan Schmid[*,†]

†*TU Wien, Institute of Sensor and Actuator Systems, Gusshausstrasse 27-29, 1040 Vienna, Austria.*

‡*Invisible-Light Labs GmbH, Taubstummengasse 11, 1040 Vienna, Austria.*

E-mail: jelena.popovic@tuwien.ac.at; silvan.schmid@tuwien.ac.at

A. Derivation of the decadic linear attenuation coefficient for sparse particle film

In the case of sparsely distributed particles with negligible optical interactions, each particle can be considered an independent absorber. For particles much smaller than the wavelength of incident light ($R << \lambda$), the absorption cross section is given by[1]:

$$\sigma_{\rm abs}(\tilde{\nu}) = 8\pi^2 \tilde{\nu} R^3 \, \mathrm{Im}\left\{ \frac{\tilde{n}^2(\tilde{\nu}) - n_{\rm m}^2(\tilde{\nu})}{\tilde{n}^2(\tilde{\nu}) + 2n_{\rm m}^2(\tilde{\nu})} \right\}, \tag{S1}$$

where $\tilde{\nu} = 1/\lambda$ is the wavenumber, $\tilde{n}(\tilde{\nu})$ the complex refractive index of the particles, and $n_m(\tilde{\nu})$ the real refractive index of the surrounding medium.

The particles sit on top of a suspended dielectric film (SiN membrane), which is optically thin compared to the wavelength λ. Hence, the refractive index of the medium is approximately that of vacuum $n_m(\tilde{\nu}) \approx 1$, resulting in:

$$\sigma_{\rm abs}(\tilde{\nu}) \approx 8\pi^2 \tilde{\nu} R^3 \, \mathrm{Im}\left\{ \frac{\tilde{n}^2(\tilde{\nu}) - 1}{\tilde{n}^2(\tilde{\nu}) + 2} \right\}. \tag{S2}$$

The decadic volume attenuation coefficient of a particle is given by:

$$\mu_{10,v}(\tilde{\nu}) = \frac{\sigma_{\rm abs}(\tilde{\nu})}{\ln(10)}, \tag{S3}$$

which can be converted to the decadic linear attenuation coefficient through division by the particle volume:

$$\mu_{10}(\tilde{\nu}) = \frac{\mu_{10,v}(\tilde{\nu})}{\frac{4}{3}\pi R^3} = \frac{6\pi\tilde{\nu}}{\ln(10)} \, \mathrm{Im}\left\{ \frac{\tilde{n}^2(\tilde{\nu}) - 1}{\tilde{n}^2(\tilde{\nu}) + 2} \right\}. \tag{S4}$$

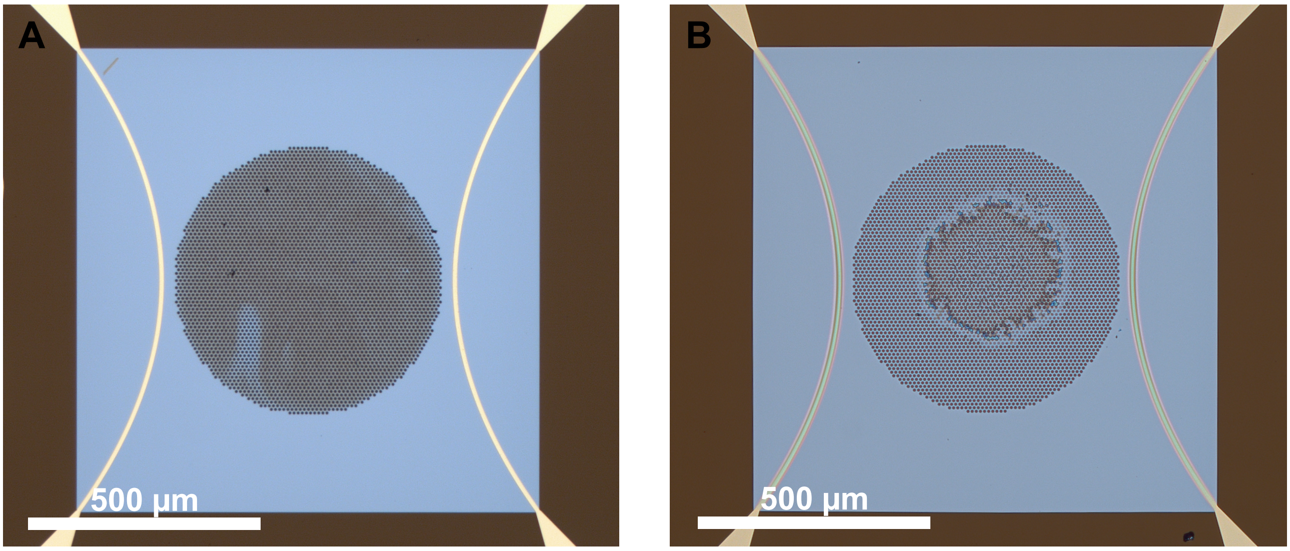

Figure S1. Sample distribution after aerosol-based deposition and drop casting in conjunction with pervaporation. (**A**) Sample distribution obtained using the aerosol-based method of deposition of the accelerated aging teabag leachates. Aerosolized particles were collected across the entire perforated area of the membrane through inertial impaction. The image corresponds to a chip sampled for 5 min at a flow rate of 0.5 L/min with teabag leachate after 5 days of accelerated aging. (**B**) A dried 500 nL droplet of the teabag leachate drop casted using micropipette and dried using the pervaporation method. The pervaporation process successfully confined the analyte towards the center of the perforated membrane.

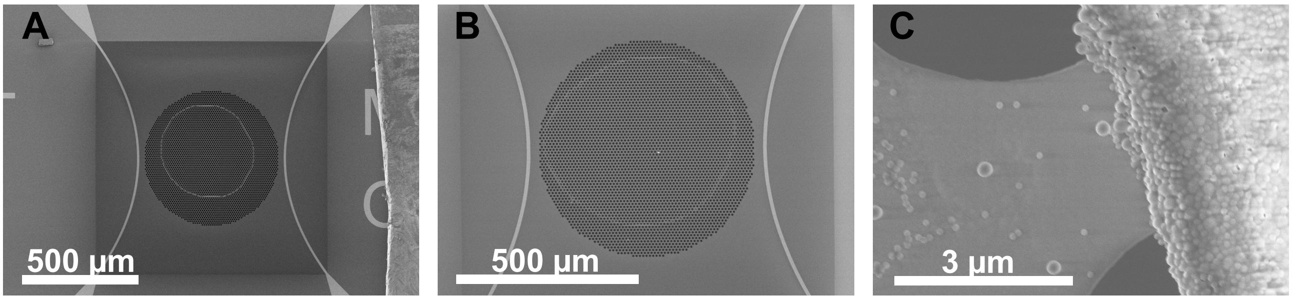

Figure S2. Sample distribution after nanodroplet dispensing. SEM image of (**A**) 1.25 ng of PP nanoparticles, (**B**) 1.25 ng of PS nanoparticles, (**C**) a PS-PP-PVC mixture mixed in a mass ratio of 1:1:1, containing 5 ng of each component, deposited onto the NEMS membrane using piezoelectric nanodroplet dispenser. The deposited samples are confined within the perforated area of the membrane following droplet drying. Images also show the coffee ring structure.

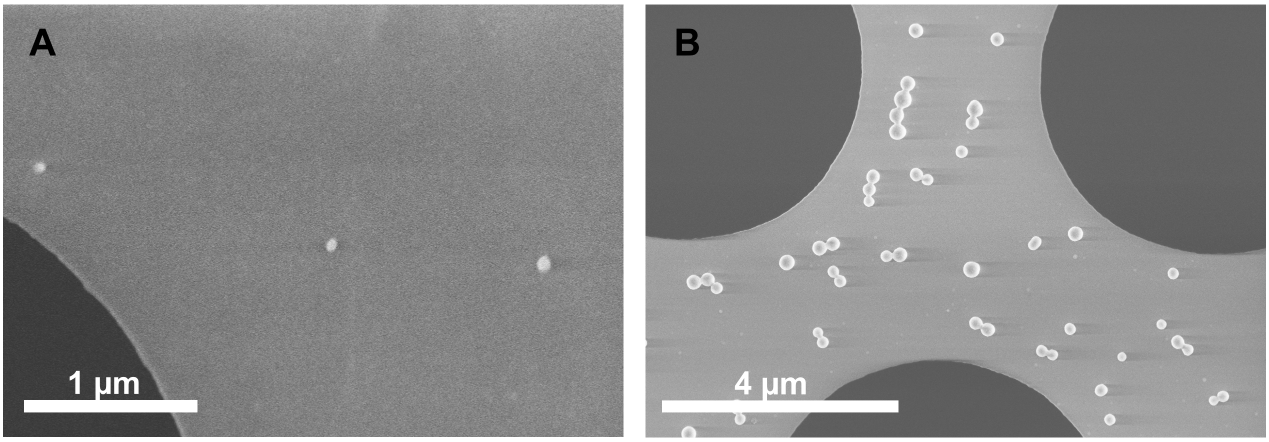

Figure S3. Nanoparticles on the perforated area of the membrane. SEM image of (**A**) PP (nominal diameter: 54 nm), and (**B**) PVC (nominal diameter: 262 nm) nanoparticles after deposition using piezoelectric nanodroplet dispenser on the perforated area of the membrane on the NEMS chip.

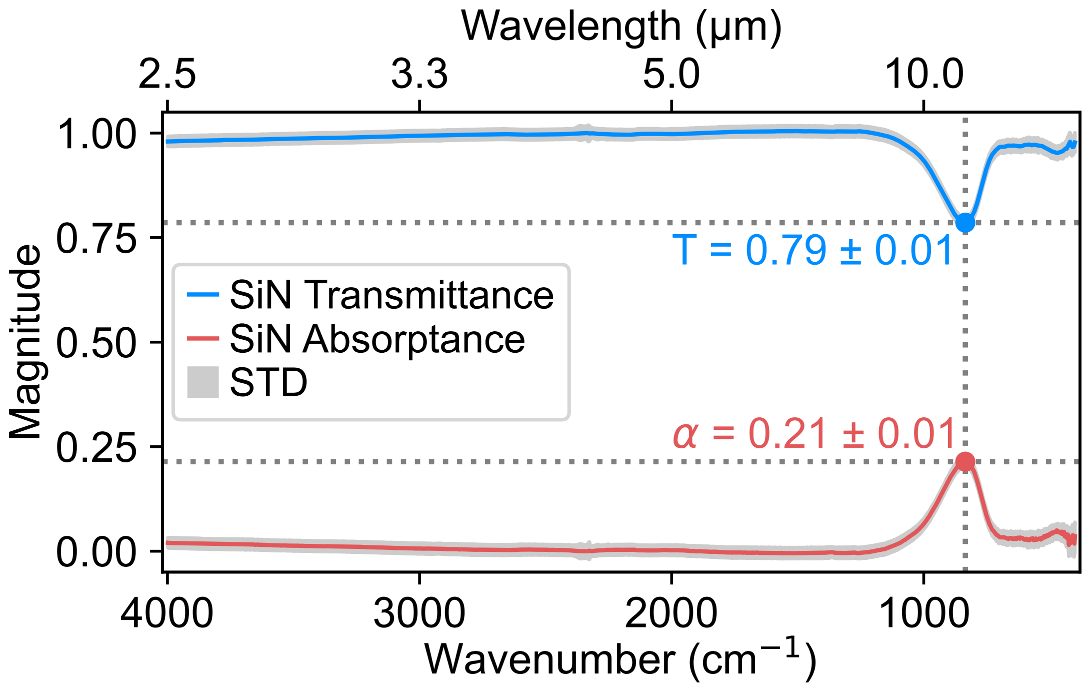

Figure S4. IR spectral features of SiN membranes. Average transmittance and absorptance spectra of 12 individual empty NEMS chips composed of 50 nm thick low-stress SiN. The shaded regions represent the standard deviations associated with each spectrum. The dashed vertical line at 835 cm^{-1} marks the characteristic SiN peak, where the transmittance and absorptance values, along with their standard deviations, were evaluated.

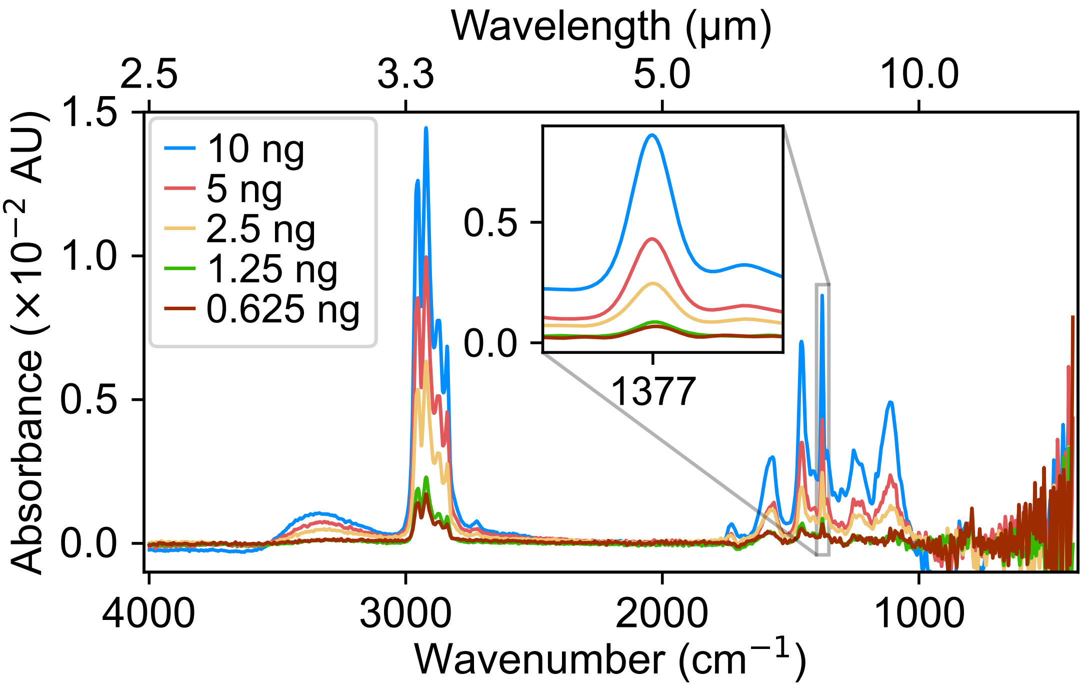

Figure S5. Characterization of PP nanoplastics. NEMS-FTIR spectra of PP nanoplastics deposited on NEMS chips at varying mass loads. The inset highlights the 1377 cm^{-1} peak, which was used to construct the calibration curve and determine the LoD.

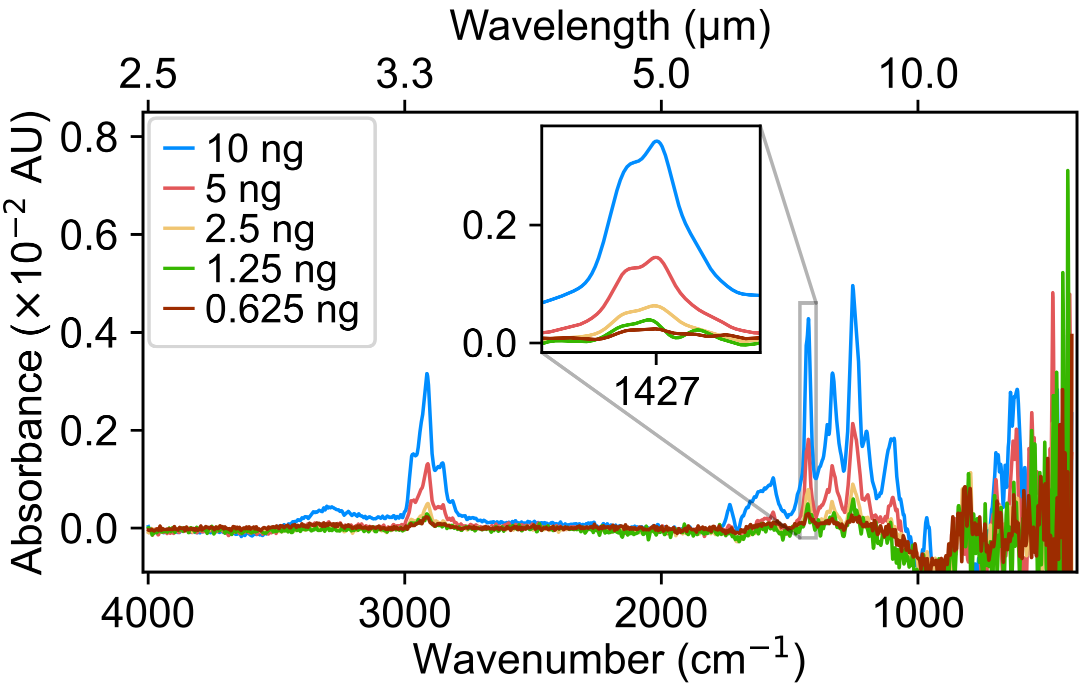

Figure S6. Characterization of PVC nanoplastics. NEMS-FTIR spectra of PVC nanoplastics deposited on NEMS chips at varying mass loads. The inset highlights the 1427 cm^{-1} peak, which was used to construct the calibration curve and determine the LoD.

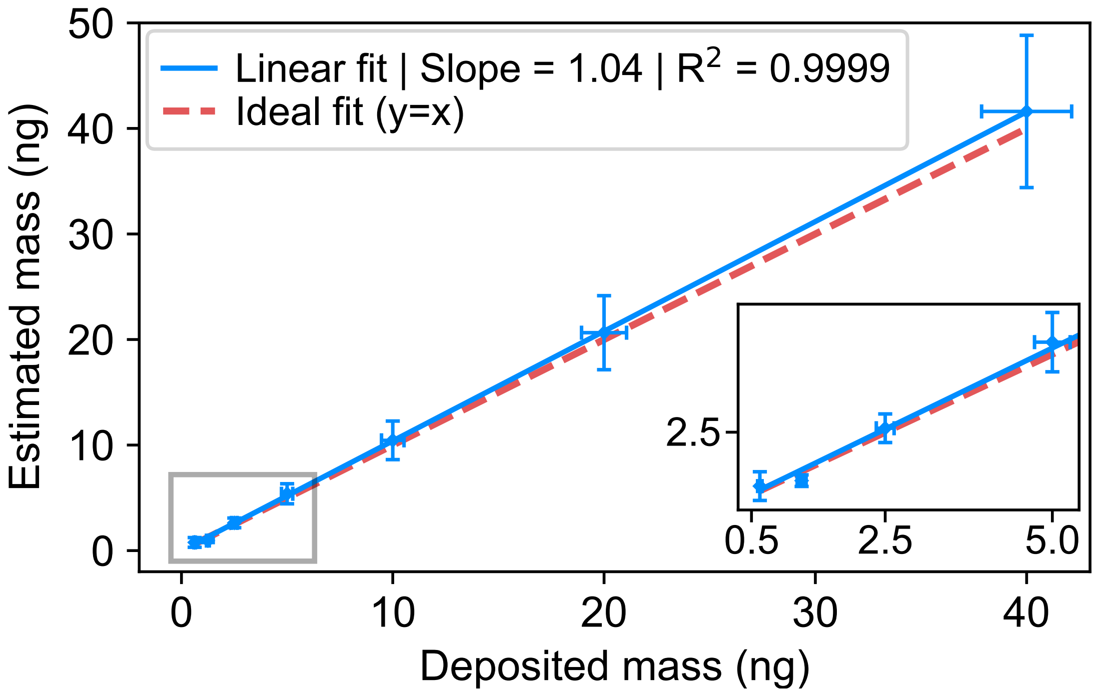

Figure S7. Quantitative analysis. Correlation between the mass of PS nanoplastic particles deposited on the NEMS chip and the estimated mass derived from the measured NEMS-FTIR spectra after conversion to optical absorbance (N=3). The inset highlights the lower mass range for improved visibility. Error bars represent propagated uncertainties from both the measurement and estimation processes.

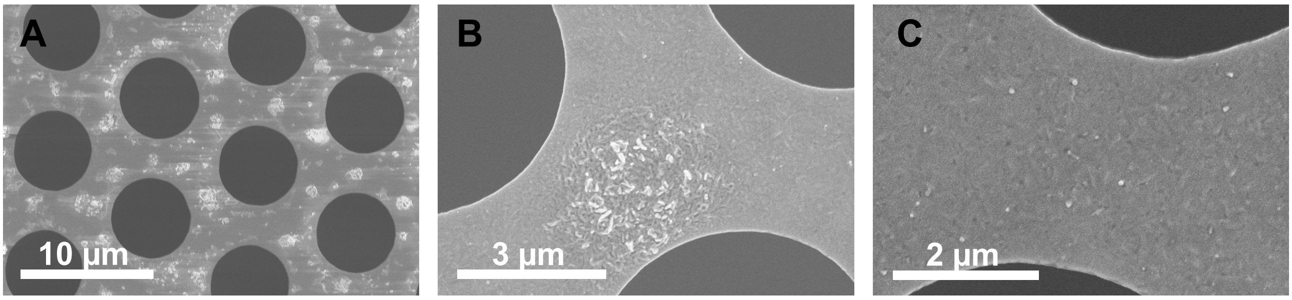

Figure S8. Micro- and nanofragments released by teabags during accelerated aging. (**A-C**) Magnified views of the NEMS membrane resonator sampled with water in which a teabag had been soaked for 15 days and exposed to environmental stress.

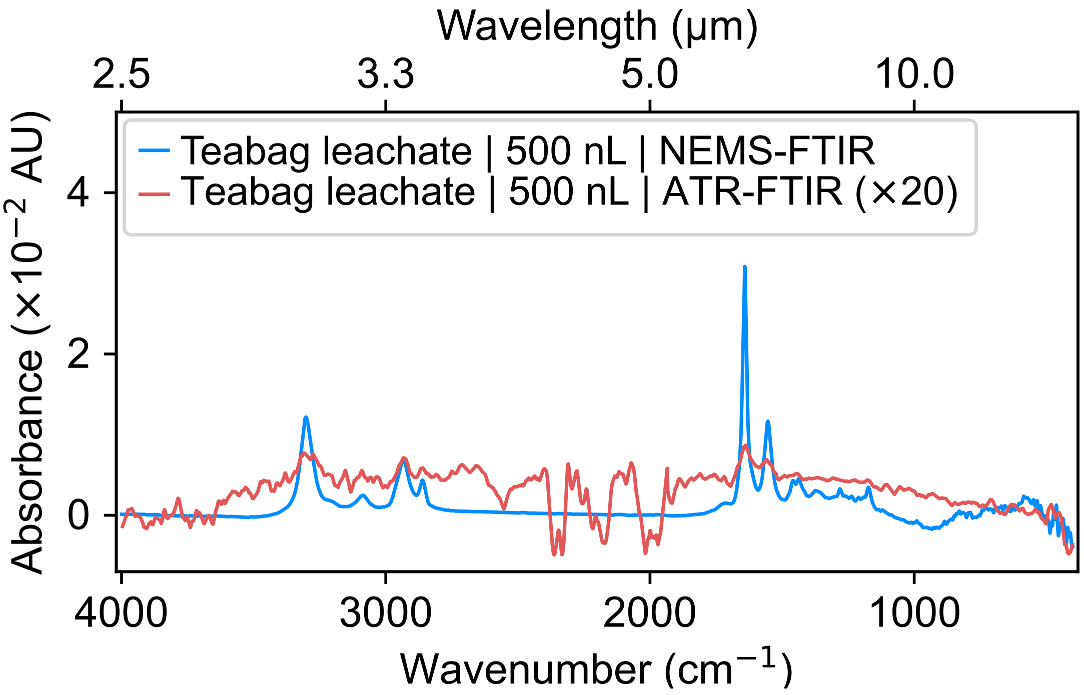

Figure S9. Comparison of NEMS-FTIR and ATR-FTIR spectra of a 500 nL teabag leachate. The ATR-FTIR spectrum of 500 nL of teabag leachate is multiplied by a factor of 20 with respect to the NEMS-FTIR spectrum of 500 nL of the same leachate for improved visibility, revealing only weak indications of the most prominent IR peaks related to nylon.

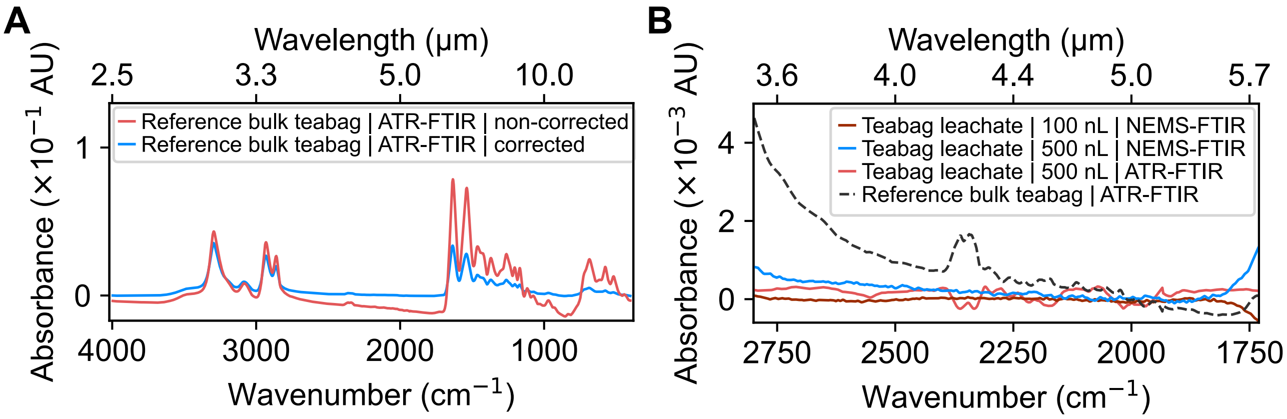

Figure S10. Correction effects and spectral artifacts in ATR-FTIR. (**A**) Comparison of non-corrected and corrected ATR-FTIR spectrum of bulk nylon teabag (reference). Corrections applied include "Extended ATR correction" (standard mode) and "Baseline correction" using OPUS software. The figure demonstrates how the applied corrections alter the relative intensities of spectral peaks. (**B**) Comparison of NEMS-FTIR spectra of 100 nL and 500 nL nylon teabag leachates with ATR-FTIR spectra of 500 nL of the same leachate and the bulk nylon teabag in the 2800–1730 cm^{-1} region. CO_2 peaks and diamond phonon bands are visible in the ATR-FTIR spectra, whereas these spectral artifacts are absent in the NEMS-FTIR spectra.

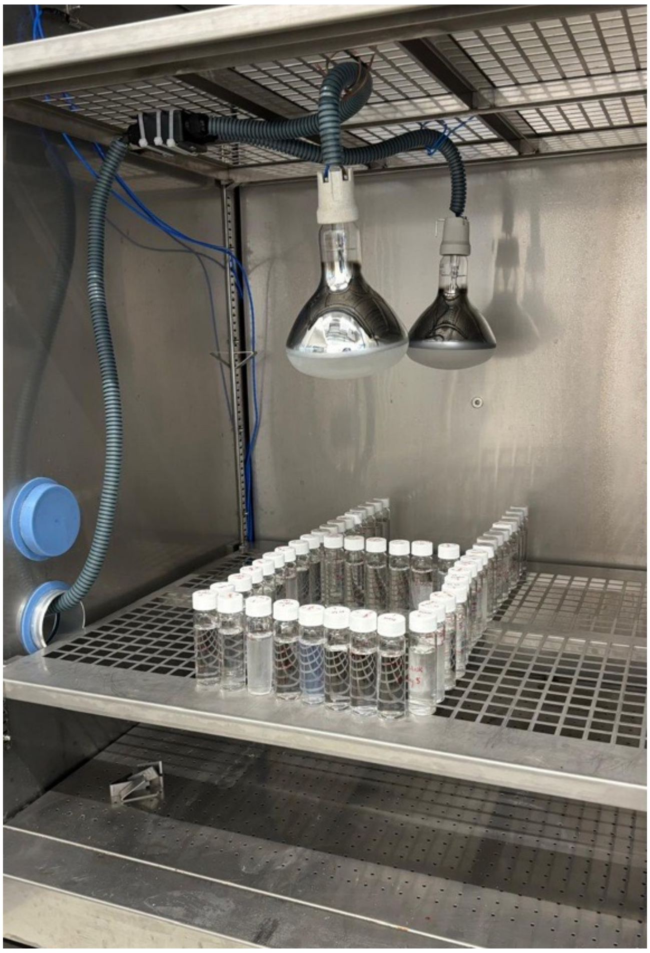

Figure S11. Experimental setup for the accelerated aging of teabags. The setup includes a controlled environment chamber for simulating environmental stress through cyclic exposure to elevated temperature and UV radiation, with teabags immersed in water for the duration of the experiment.

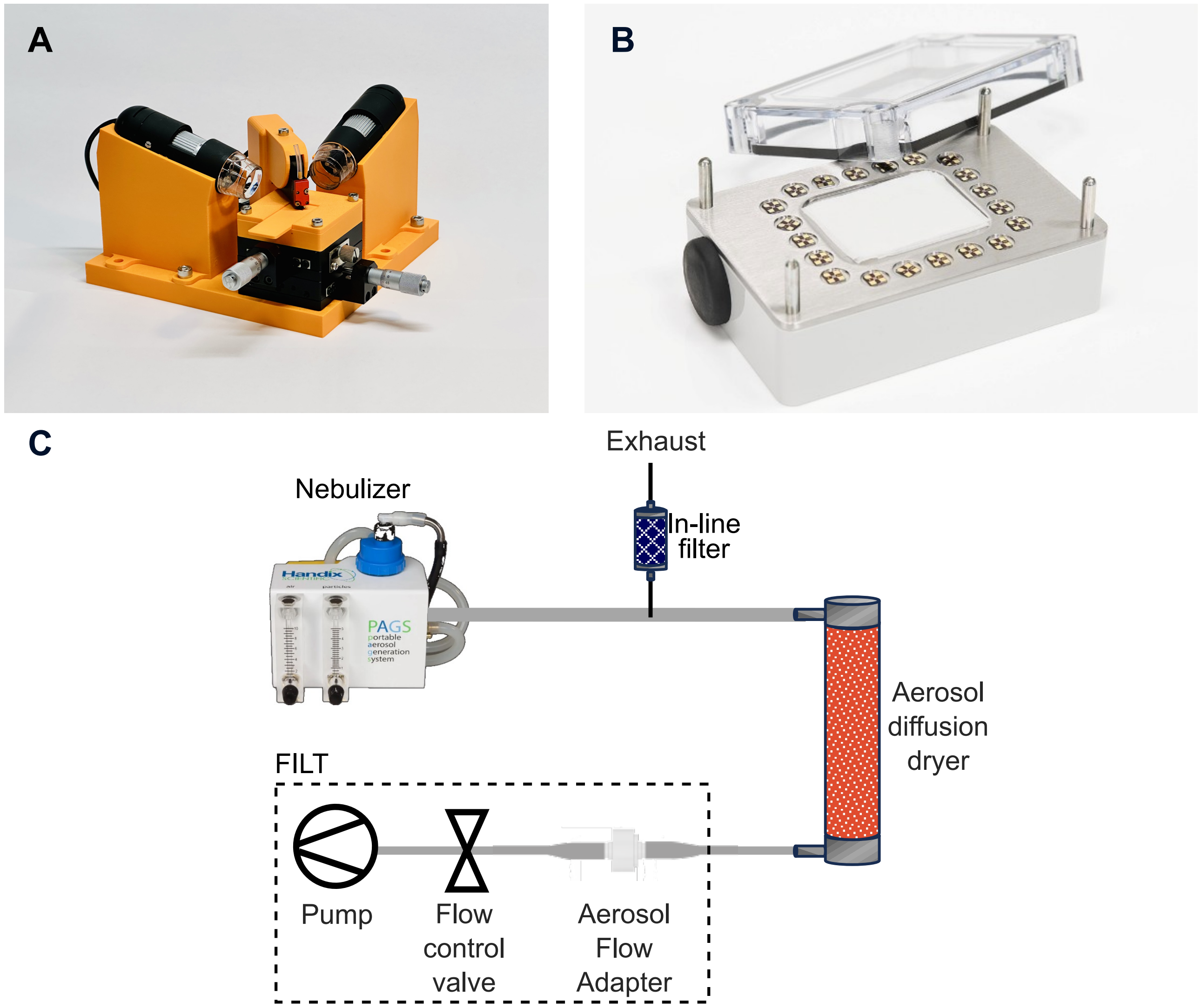

Figure S12. Sampling setups. (**A**) The nanodroplet dispensing set-up consisted of the piezoelectric nanodroplet dispenser (PIPEJET nanoDispenser, BioFluidix-Hamilton Freiburg, Germany) equipped with two cameras (USB Mikroskop, Prod. No. TO-5139591, TOOLCRAFT, Germany) and the XY stage (XY Axis Manual Displacement Platform, Prod. No. B07TQRWQH4, Amazon, Germany) mounted using a custom 3D-printed scaffold. The set-up enabled precise positioning of the NEMS chips beneath the dispensing capillary for accurate droplet deposition onto the central region of the perforated membrane area. (**B**) The Drop Casting Accessory (Invisible-Light Labs GmbH, Austria) for liquid samples. The accessory allows for precise sample deposition and controlled drying under a humidity gradient *via* pervaporation, ensuring consistent localization of the sample within the perforated membrane area. (**C**) Schematic representation of the setup, comprising a nebulizer (Portable Aerosol Generation System, PAGS, Handix Scientific Inc., CO, USA) for aerosolizing the liquid into fine droplets, a diffusion dryer (DDU, 570/L, Topas GmbH, Germany), and a NEMS chip holder (Aerosol Flow Adapter, Invisible-Light Labs GmbH, Austria) containing the NEMS chip. The chip holder was placed in an eight-channel filter sampler (FILT, Brechtel Manufacturing Inc., CA, USA), which includes a built-in pump. Sampling is based on the inertial impaction deposition mechanism, enabling sample deposition across the entire perforated membrane area.

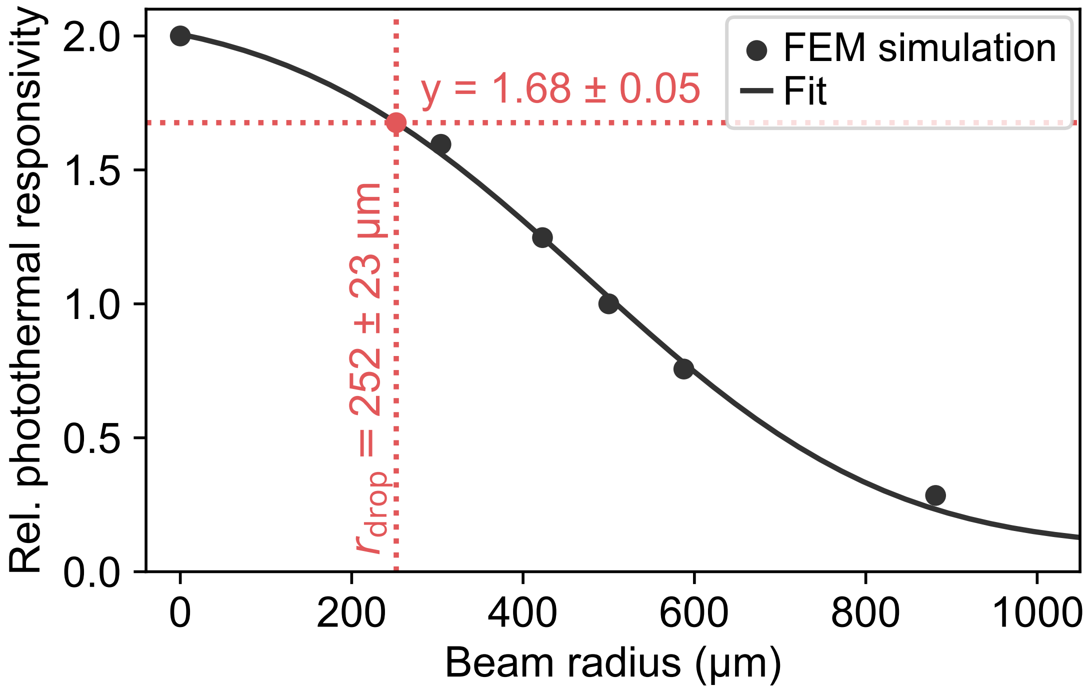

Figure S13. Dependence of relative photothermal responsivity on the IR beam radius. Data obtained through FEM simulations of a 1x1 mm^2 low-stress SiN membrane. The graph highlights how varying the beam radius affects the responsivity of the NEMS membrane due to spatial heat distribution.

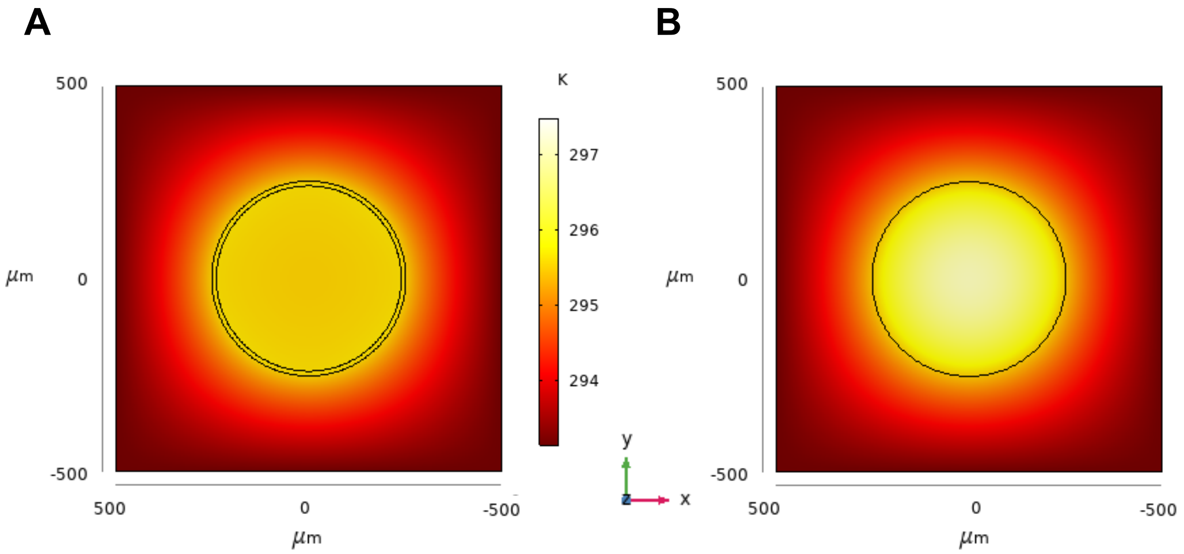

Figure S14. Temperature distribution in a SiN membrane induced by laser heating. (A) Simulated temperature distribution on a 1×1 mm^2 SiN membrane resulting from a ring-shaped heat source (diameter 252 μm), mimicking a donut-shaped laser beam. **(B)** Temperature distribution on the same membrane with a disk-shaped heat source of the same diameter. The difference in photothermal optomechanical response between these distributions is approximately 3%.

Table S1. Characteristic IR absorption bands of plastics. Overview of the main IR absorption peaks identified in the NEMS-FTIR spectra for PS, PP, PVC, and PA (nylon) particles, together with corresponding band assignments. The cited references originate from transmission and ATR-FTIR measurements, demonstrating direct comparison between NEMS-FTIR and established IR spectroscopy data.

Type of plastics	Peak position (cm^{-1})	Assignment	References
PS	700	Aromatic ring bending	2–5
	753	Aromatic out-of-plane C–H bending	2–5
	1028	C–H in-plane bending of the phenyl ring	3,4,6
	1452	C–H bending (scissoring, aliphatic backbone)	3–6
	1493		2–6
	1583	Aromatic C–C stretching (ring vibration)	3,4
	1601		2–6
	1745		2,4
	1800	Aromatic overtone/combination band	2–4
	1868	(benzene fingers)	2–4
	1943		2–4
	2849	Aliphatic C–H stretching (CH_2	2–5
	2923	asymmetric and symmetric)	2–5
	3026		2–5
	3060	Aromatic C–H stretching	2–4
	3081		2–4
PP	1115	C–C chain stretching + CH_3 rocking + CH_2 wagging + CH twisting + CH bending	7
	1167	C–H bending + C–C chain stretching + CH_3 rocking	6–8
	1220	CH_2 twisting + CH bending + C–C chain stretching	7
	1254	CH bending + CH_2 twisting + CH_3 rocking	7
	1377	CH_3 bending, symmetric (umbrella mode)	6,8,9

Type of plastics	Peak position (cm^{-1})	Assignment	References
	1459	CH_3 bending, symmetric	6,8,9
	2839	CH_2 stretching, symmetric	2,8
	2868	CH_3 stretching, symmetric	2,8,9
	2920	CH_2 stretching, asymmetric	2,8,9
	2952	CH_3 stretching, asymmetric	2,8,9
PVC	614	C-Cl stretching	6,10
	637		6,10
	692		6,10,11
	970	CH_2 rocking	6,8,10,11
	1100	C-C stretching	8,10,11
	1254	C-H bending, in-phase	8,10,11
	1334	C-H bending, out-of-phase	8,10–12
	1427	CH_2 bending, in-phase	8,10,11
	1625	C=C stretching	12
	2849	CH_2 stretching, symmetrical, in-phase	11
	2910	C-H stretching of CH_2, asymmetrical, in-phase	8,10,11
	2971	C-H stretching of CHCl, out-of-phase	10,11
PA	575	C=O out-of-plane vibration (amide VI)	13,14
	990	C-CO stretching (amide IV)	13–15
	1116	C-N stretching	16,17
	1170	C-C stretching	13
	1210	CH_2 wagging or twisting	13
	1235	C-N stretching	17
	1282	C-N stretching + CH_2 twisting	13,17
	1371	CH_2 wagging + C-N-H deformation	13–15

Type of plastics	Peak position (cm^{-1})	Assignment	References
	1437	CH_2 (Gauche bonds) deformation	13
	1460	CH_2 (adjacent to N-H group) deformation	13,14
	1553	N-H in-plane bending + C-N stretching (amide II)	13,14,17
	1642	C=O stretching (amide I)	13,14,17
	1720	Imide group; carboxylic acid C=O stretching mode	15,18,19
	2860	C-H stretching, symmetrical	13,14
	2930	C-H stretching, asymmetrical	13,14
	3087	C-N stretching + overtone of N-H in-plane bending	13–15,17
	3300	N-H stretching	8,13,14,16,17

Table S2. Deposited and estimated mass of PS NPs. Quantitative comparison of the deposited and estimated mass values for PS nanoparticles on NEMS chips, accompanied by their respective standard deviations. Deposited masses were derived from the dispersion concentrations and droplet volume, with uncertainties propagated from sample preparation (micropipette) and deposition tools (piezoelectric nanodroplet dispenser). Estimated masses were calculated using Eq. (6). The main source of uncertainty in estimated mass originates from variability in the measured absorptance at 1452 cm^{-1}.

**Deposited mass (ng)**	**SD (ng)**	**Estimated mass (ng)**	**SD (ng)**
0.62	0.03	0.8	0.5
1.2	0.1	1.0	0.2
2.5	0.1	2.6	0.5
5.0	0.3	5	1
10.0	0.5	10	2
20	1	21	4
40	2	42	7

References

1. Baffou, G.; Quidant, R. Thermo-Plasmonics: Using Metallic Nanostructures as Nano-Sources of Heat. *Laser Photonics Rev.* **2012**, *7*, 171–187.

2. Smith, B. C. The Infrared Spectra of Polymers III: Hydrocarbon Polymers. *Spectroscopy* **2021**, 22–25.

3. Wu, B.; Wu, X.; Liu, S.; Wang, Z.; Chen, L. Size-Dependent Effects of Polystyrene Microplastics on Cytotoxicity and Efflux Pump Inhibition in Human Caco-2 Cells. *Chemosphere* **2019**, *221*, 333–341.

4. Myers, T. L.; Tonkyn, R. G.; Oeck, A. M.; Danby, T. O.; Loring, J. S.; Taubman, M. S.; Sharpe, S. W.; Birnbaum, J. C.; Johnson, T. J. In *NIST Chemistry WebBook, NIST Standard Reference Database Number 69*; Linstrom, P. J., Mallard, W. G., Eds.; National Institute of Standards and Technology: Gaithersburg, MD, USA, 2025; retrieved January 24, 2025.

5. Zając, M.; Kotyńska, J.; Worobiczuk, M.; Breczko, J.; Naumowicz, M. The Effect of Submicron Polystyrene on the Electrokinetic Potential of Cell Membranes of Red Blood Cells and Platelets. *Membranes* **2022**, *12*, 366.

6. Böke, J. S.; Popp, J.; Krafft, C. Optical Photothermal Infrared Spectroscopy with Simultaneously Acquired Raman Spectroscopy for Two-Dimensional Microplastic Identification. *Sci. Rep.* **2022**, *12*, 18785.

7. Karacan, İ.; Benli, H. The Use of Infrared-Spectroscopy Technique for the Structural Characterization of Isotactic Polypropylene Fibres. *Tekstilvekonfeksiyon* **2011**, *21*, 116–123.

8. Tagg, A. S.; Harrison, J. P.; Ju-Nam, Y.; Sapp, M.; Bradley, E. L.; Sinclair, C. J.; Ojeda, J. J. Fenton's Reagent for the Rapid and Efficient Isolation of Microplastics from Wastewater. *Chem. Commun.* **2017**, *53*, 372–375.

9. Fang, J.; Zhang, L.; Sutton, D.; Wang, X.; Lin, T. Needleless Melt-Electrospinning of Polypropylene Nanofibres. *J. Nanomater.* **2012**, *2012*, 382639.

10. Beltran, M.; Marcilla, A. Fourier Transform Infrared Spectroscopy Applied to the Study of PVC Decomposition. *Eur. Polym. J.* **1997**, *33*, 1135–1142.

11. Stromberg, R.; Straus, S.; Achhammer, B. Infrared Spectra of Thermally Degraded Poly(vinylchloride). *J. Res. Natl. Bur. Stand.* **1958**, *60*, 147.

12. Fawzi, S.; Yousif, E.; Zainulabdeen, K.; Bufaroosha, M.; Ahmed, D. Highly Effective Photostabilization of Polyvinyl Chloride Films Using Omeprazole-Tin Additive Complexes. *J. Umm Al-Qura Univ. Appl. Sci.* **2025**, 1–25.

13. Kang, H.; Wang, Z.; Hao, X.; Liu, R. Thermal Induced Crystalline Transition of Bio-Based Polyamide 56. *Polymer* **2022**, *242*, 124540.

14. Tummino, M. L.; Chrimatopoulos, C.; Bertolla, M.; Tonetti, C.; Sakkas, V. Configuration of a Simple Method for Different Polyamides 6.9 Recognition by ATR-FTIR Analysis Coupled with Chemometrics. *Polymers* **2023**, *15*, 3166.

15. Gonçalves, E. S.; Poulsen, L.; Ogilby, P. R. Mechanism of the Temperature-Dependent Degradation of Polyamide 66 Films Exposed to Water. *Polym. Degrad. Stab.* **2007**, *92*, 1977–1985.

16. Shurvell, H. Spectra– Structure Correlations in the Mid- and Far-Infrared. *Handbook of Vibrational Spectroscopy* **2001**,

17. Smith, B. C. Infrared Spectroscopy of Polymers, XI: Introduction to Organic Nitrogen Polymers. *Spectroscopy* **2023**, 14–18.

18. Zimudzi, T. J.; Feldman, K. E.; Sturnfield, J. F.; Roy, A.; Hickner, M. A.; Stafford, C. M. Quantifying Carboxylic Acid Concentration in Model Polyamide Desalination Membranes via Fourier Transform Infrared Spectroscopy. *Macromolecules* **2018**, *51*, 6623–6629.

19. Okamba-Diogo, O.; Richaud, E.; Verdu, J.; Fernagut, F.; Guilment, J.; Fayolle, B. Molecular and Macromolecular Structure Changes in Polyamide 11 During Thermal Oxidation. *Polym. Degrad. Stab.* **2014**, *108*, 123–132.
